# GPs’ prescription patterns, experience, and attitudes towards medicinal cannabis—a nationwide survey at the early stage of the Danish test scheme

**DOI:** 10.1186/s12875-023-01971-4

**Published:** 2023-01-17

**Authors:** F. Rosenbæk, H. Riisgaard, J. B. Nielsen, S. Wehberg, F. B. Waldorff, L. B. Pedersen, J. Søndergaard

**Affiliations:** 1 Research Unit of General Practice, Department of Public Health, University of Southern Denmark, J.B. Winsløws Vej 9A, Odense C, 5000 Denmark; 2 The Research Unit for General Practice and Section of General Practice, Department of Public Health, University of Copenhagen, Copenhagen, Denmark; 3 Department of Public Health, DaCHE – Danish Centre for Health Economics, University of Southern Denmark, Winsløws Vej 9B, Odense C, 5000 Denmark

**Keywords:** Medical cannabis, General practitioner, Prescription, Attitude, Experience, Survey, Questionnaire

## Abstract

**Background:**

On 1 January 2018 a four-year test scheme concerning use of medicinal cannabis (MC) was enacted. It has recently been extended for four more years by the Danish Parliament permitting all Danish physicians to prescribe MC to their patients. Previous studies have shown that general practitioners (GPs) have varying prescription experience, little knowledge, and mixed attitudes about MC. However, the present evidence is still limited, and no studies exist about Danish GPs’ prescription experience, knowledge, and attitudes towards MC. Therefore, our aim was to examine Danish GPs’ prescription experience, knowledge, and attitudes towards MC.

**Methods:**

A national online survey-based study addressing Danish GPs was performed from September 2018 to July 2019. We performed separate multivariable logistic regression analyses including GPs’ prescription experience, knowledge, and attitudes towards MC as outcome variables.

**Results:**

A total of 427 (38.4%) of 1112 GPs completed the questionnaire. Of these, 37 (8.7%) had experience in prescribing MC. The majority had little or no knowledge about MC (80.6%) as well as a negative view on prescription of MC (71.4%) to patients. Factors associated with prescribing MC to patients were: Single-handed practices (OR = 1.6, 95% CI 1.1;1.8) and perception of having quite some knowledge about MC (OR = 4.8, 95% CI 2.2;10.4). Factors associated with having quite some knowledge about MC were: having a positive attitude towards prescribing MC (OR = 5.2, 95% CI 1.9;14.0), being male (OR = 1.7, 95% CI 1.4;1.8), and being at least 60 years of age (OR = 2.8, 95% CI 1.3;6.0). Factors associated with having a positive attitude towards prescribing MC were: having quite some knowledge about MC (OR = 5.2, 95% CI 2.2;12.5) and GPs being male (OR = 1.7, 95% CI 1.1;1.9).

**Conclusion:**

In this first study on prescription experience, knowledge, and attitudes about MC among Danish GPs, conducted one year after the Danish test scheme was enacted, we find a very low proportion of prescribers, little knowledge, and an overall negative attitude towards MC. Among the prescribing GPs, four in ten have little to no knowledge and a negative attitude towards MC. We stress that prescribing patterns, knowledge, and attitudes may change throughout the remaining time of the test scheme.

**Supplementary Information:**

The online version contains supplementary material available at 10.1186/s12875-023-01971-4.

## Background

In recent years, there have been extensive discussions in western countries whether to legalize cannabis for medicinal purposes and the attitudes among legislators in several of those countries now seem to be shifting towards controlled legalization [1–3]. The arguments presented by legislators include scientific evidence indicating possible associations between medicinal cannabis (MC) use and pain relief of spasms and neuropathy, and control of nausea and vomiting [4]. This is also the case in Germany, as German physicians are most likely to prescribe for the indications of pain and spasticity [5]. It is, however, widely argued by physicians that the limited evidence on both efficacy and potential adverse effects calls for further investigations before legalizing cannabis as a medical treatment [6–8]. The medical societies in Denmark have opposed legalizing MC due to lack of solid evidence of effects and side effects, as well as due to legal issues on treatment responsibilities. As for most other medical products, Danish general practitioners (GPs) are expected to handle the major part of MC prescriptions. However, studies have shown that many GPs are concerned about the lack of sufficient evidence for legalizing the use of MC [9–11]. The GPs who are not familiar with prescribing MC express worries about their own knowledge and training [12], and are not comfortable with prescribing MC unless their peers agree [9]. GPs and hospital physicians experience frequent enquiries about MC, but not all studies display accordingly high proportions of GPs who have ever prescribed it [13].

The overall aim of this study is to explore GPs’ decision to prescribe MC to patients and patterns of knowledge and attitudes towards MC in a Danish setting. Accordingly, the primary objective of the study is to explore the associations between 1) GPs’ experience with prescribing MC to patients and GP and practice characteristics. Secondarily, we want to explore 2) GPs’ knowledge about MC in relation to GP and practice characteristics and 3) GPs’ attitudes towards MC and associations with GP and practice characteristics.

## Methods

### Setting

Denmark is a country with around 5.9 million inhabitants where the healthcare is primarily publicly funded through taxes [14]. Approximately 98% of Danish citizens are listed with a GP. There are approximately 3300 GPs in a total of 1635 practices and GPs are self-employed, but work on a contract with the public funder [15]. GPs are responsible for the primary care needs of their listed patients. They act as gatekeepers to the health care system, controlling access to most primary care specialists as well as in- and outpatient hospital care through a referral system [16, 17].

On 1 January 2018, the Danish Parliament enacted a four-year test scheme concerning prescription and use of MC [18]. This was recently extended by 1 January 2022 until 2026 [19]. The scheme permits all Danish physicians to prescribe MC to their patients, and The Danish Medicines Agency listed the following four indications for prescribing: 1) painful spasms caused by multiple sclerosis, 2) painful spasms due to spinal cord injury, 3) nausea after chemotherapy, and 4) neuropathic pain that is pain due to disease of the brain, spinal cord, or nerves [19]. However, the physicians had the legal right to prescribe MC to any indication. They also had the legal responsibility, meaning that they were held accountable, if the prescribed MC turned out to be harmful for the patients [18, 19]. Recent data from Danish registries suggest that patients who fall into aforementioned indications 1) and 2) are the most prominent users under the Danish test scheme [19, 20].

### Study design and data collection

A database was made uniquely for this study to gather information about all GPs in Denmark, by combining data from two national registries [21, 22]. This made it possible to draw a random sample of one third of all Danish GPs from all five regions. The GPs received a postal invitation to participate in an electronic survey performed between September 2018 and July 2019. To account for the varying number of GPs across the five Danish regions, we stratified the sample according to one third in each of the five regions rather than sampling on a national level. Two reminders followed the invitation approximately three and six weeks after, respectively.

### Questionnaire development

The questionnaire was developed for physicians, including GPs.

First, a systematic literature search was conducted uncovering central themes in relation to physicians’ attitudes and knowledge about MC, and motives for prescribing or not prescribing MC and/or cannabis-based medicine (CBM). MC is defined as dried plant parts or extracts of plant parts (cannabis oils) of the stem plant Cannabis Sativa. CBM refers to extracts of the stem plant Cannabis Sativa or synthetically produced cannabinoids [23].

Second, interviews with nine strategically selected physicians in relevant specialties were conducted to further qualify the themes to be included in the questionnaire. Four of the physicians had family medicine as their specialty, one of them worked as a pain specialist, and the rest of them were GPs. This selection was done to ensure the widest possible representation of relevant medical specialties.

Third, the themes to be included as questions in the questionnaire were agreed upon in the research group, and a first version of the questionnaire was developed. A total of 33 items were included and distributed on the four domains: “experiences”, “knowledge”, “attitudes”, and “GP and practice characteristics”. After each of the domains, a text box was inserted allowing the respondents to make comments to the questionnaire or to elaborate their answers.

Fourth, a total of five physicians in various relevant specialties completed a qualitative pilot test. An oncologist, a neurologist, a GP, and two anaesthesiologists of whom one had family medicine as specialty. They tested relevance, acceptability, and feasibility as well as comprehensibility and completeness of the questionnaire. Eight items were added, and a few other changes were made primarily to meet comments on comprehensibility and relevance.

Fifth, after incorporating the extra items and their relevant comments into the questionnaire, a quantitative pilot test was conducted by 12 physicians – six GPs, one physician with specialty in family medicine currently working as a specialist in general surgery and gastrointestinal surgery, two anaesthesiologists, two neurologists, and one oncologist.

### The questionnaire

The questionnaire encompassed 41 items at the final stage divided into four parts. A part focusing on attitudes towards MC, a part exploring knowledge of MC, a part concerning the physicians’ experiences with prescription of MC and CBM, and a part made for collecting personal data on the respondents, including physician and practice characteristics.

In the part concerning experiences, the physicians were asked if they had been approached by patients regarding use of MC or CBM, and whether they had ever prescribed it, and if so, on what indications. In the part about knowledge, the questions concerned overall knowledge in relation to prescription behaviour and specific knowledge about indications, dosage, and side effects, among others. In the part focusing on attitudes, they were asked about their overall attitude towards MC as well as several specific uncovering questions, for instance about evidence, legislation, and patient safety. In the fourth part concerning personal data, they were asked to report sex, age, and specialty, among others. Physicians being specialists in general practice were additionally asked about their practice type (see Appendix file 1).

### Outcomes

We investigated one primary outcome “prescription of MC” and two secondary outcomes, “knowledge about MC”, and “attitudes towards MC”. The first outcome measure used three categories: prescription of MC, prescription of CBM, and not prescribing.

These categories were dichotomized into “At least one prescription of MC” and “No prescription of MC”. Prescribing CBM falls under “No prescription of MC”, as MC is the only medicine of interest in this study.

The other two outcome measures used a six-point Likert scale. For the question on overall knowledge, the categories were “To a very high extent”, “To a high extent”, “Somewhat”, “To a lesser extent”, “Not at all”, and “Do not know/not relevant”. These categories were dichotomized for analytical purposes into “Quite some knowledge” and “Little to no knowledge”, with the first one covering “To a very high extent”, “To a high extent” and “Somewhat”, and the second one covering “To a lesser extent”, “Not at all”, and “Do not know/not relevant”. For the question on attitudes towards MC, the categories were “Very positive”, “Predominantly positive”, “Neither positive nor negative”, “Predominantly negative”, “Very negative”, and “Do not know/not relevant”. For analytical purposes, those categories were grouped into “Positive attitude”, “Neither positive nor negative”, and “Negative attitude”, with the first one covering “Very positive” and “Predominantly positive”, the second one covering “Neither positive nor negative”, and the third one covering “Predominantly negative”, “Very negative”, and “Do not know/not relevant”.

### Statistical analyses

The statistical analyses were performed in two steps. Initially, we performed a non-respondent analysis using Chi-squared tests to compare respondents and non-respondents by sex (male/female), age (30–44/45–59/60 +), and region of residence (North-/Central-/Southern-/Zealand-/Capital Region of Denmark). Second, separately for the three outcomes, that is prescribing MC, knowledge about MC, and attitudes towards MC, we performed both univariable and multivariable logistic regression analyses estimating odds ratios and corresponding 95% confidence intervals, to investigate associations between practice and GP characteristics, and the three outcomes. The explaining variables were sex (male/female), age (30–44/45–59/60 +), and practice type (partnership/singlehanded). Throughout the analyses a *p*-value below or equal to 0.05 was considered statistically significant. All analyses were performed in Stata version 16 [24].

## Results

### Descriptive results

A total of 1112 postal invitations to participate in the survey were sent out to the GP sample, and 427 responded to the questionnaire (38.4%). The respondent analysis showed an overall resemblance to the entire study population concerning sex. However, we found an overrepresentation of respondents among the youngest age groups (30–44 and 45–59 years), and from The Region of Southern Denmark and The Central Denmark Region (see Table 1).

**Table 1:** Respondent analysis

	**Non-respondents N (%)**	**Respondents** **N (%)**	**Total** **N (%)**	***P*-value, χ**^**2**^**-test**
**Total**	685 (100.0)	427 (100.0)	1112 (100.0)	
**Age**				0.02*
30–44	136 (19.85)	121 (28.34)	257 (23.11)	
45–59	296 (43.21)	217 (50.82)		
60+	167 (24.38)	89 (20.84)	256 (23.02)	
Missing	86 (12.55)			
**Gender**				0.82
Female	374 (54.60)	236 (55.27)	610 (54.86)	
Male	311 (45.40)	191 (44.73)	502 (45.14)	
**Region**				0.00*
The North Denmark Region	54 (7.88)	21 (4.92)	75 (6.74)	
Central Denmark Region	129 (18.83)	140 (32.79)	269 (24.19)	
The Region of Southern Denmark	143 (20.88)	118 (27.63)	261 (23.47)	
The Capital Region of Denmark	253 (36.93)	98 (22.95)	351 (31.56)	
Region Zealand	106 (15.47)	50 (11.71)	156 (14.03)	

^*^Statistically significant at a *p*-value level ≤ 0.05

**Fig. 1:**
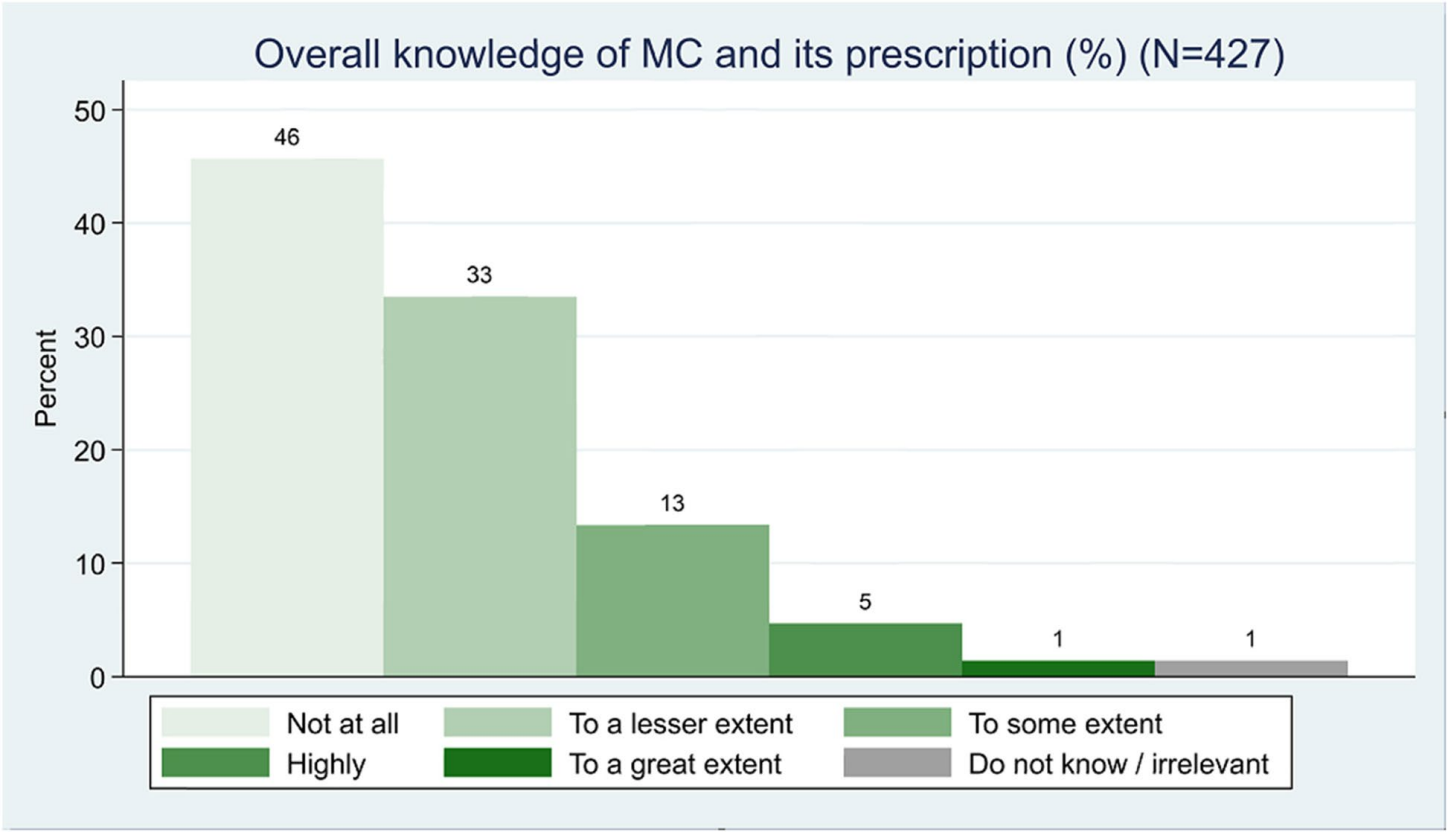
Overall knowledge of MC and its prescription

**Fig. 2:**
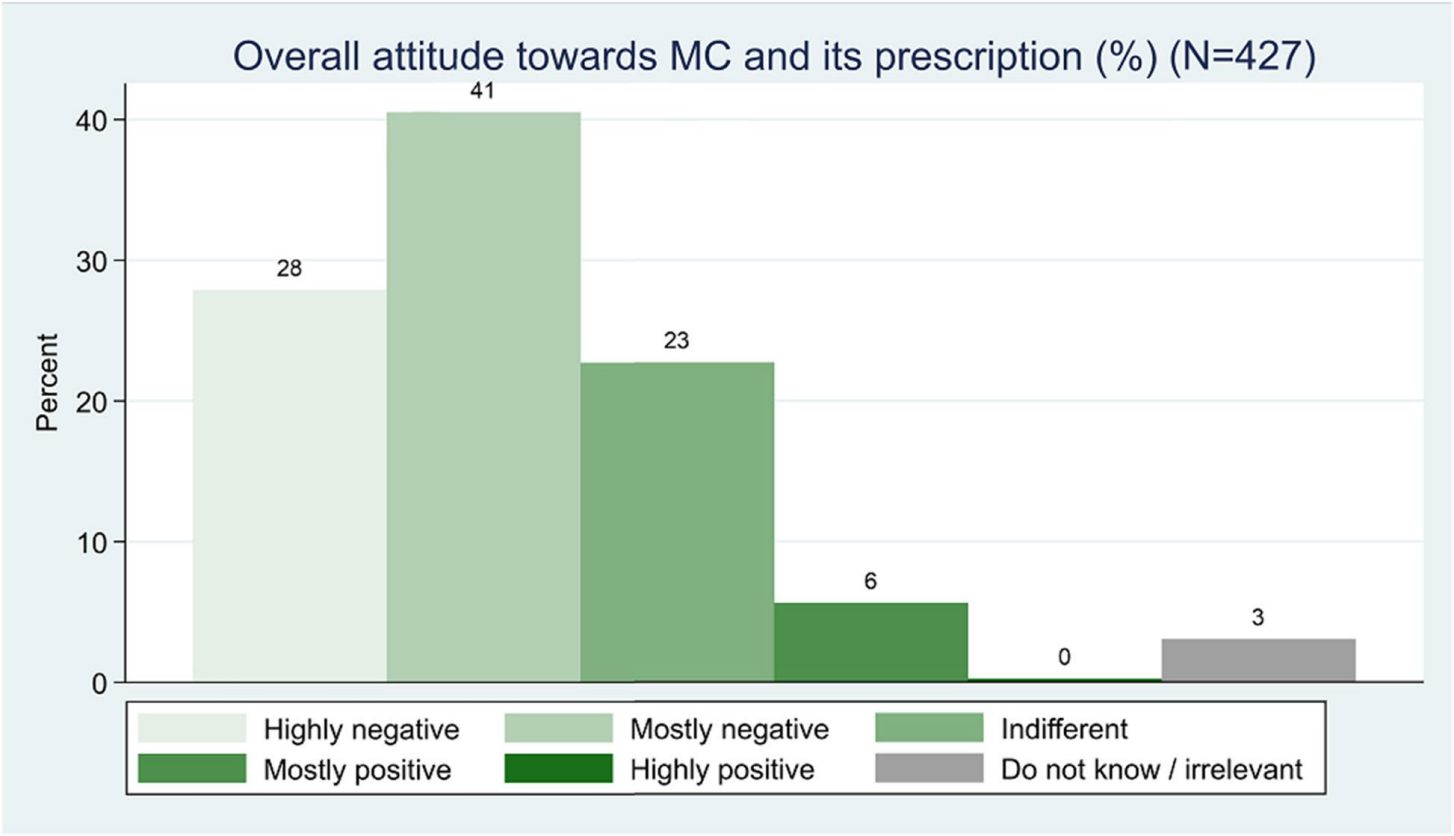
Overall attitude towards MC and its prescription

Among the responding GPs, only 37 GPs (8.7%) answered that they had prescribed MC for one or more patients with indications as stated in guidelines for the national test scheme. 83 GPs (20%) stated to have quite some knowledge of MC and its use, 25 GPs (5.8%) were positive towards MC in general, and 97 GPs (22.7%) were neither positive nor negative towards it (see Table 2, Figs. 1 and 2).

**Table 2:** Associations between practice and GP characteristics and GPs’ prescription of medicinal cannabis

	**All**	**At least one prescription of medicinal cannabis**	**Univariable regression**	**Multivariable regression**
	427 (100.0)	37 (100.0)		
**Practice type**				
Partnership	94 (22.0)	17 (45.9)	1	1
Singlehanded	333 (78.0)	20 (54.1)	1.7 (1.4;1.9)*	1.6 (1.1;1.8)*
**Gender**				
Female	236 (55.3)	11 (29.7)	1	1
Male	191 (44.7)	26 (70.3)	1.7 (1.4;1.9)*	1.4 (0.5;1.7)
**Age**				
30–44	121 (28.3)	5 (13.5)	1	1
45–59	217 (50.8)	17 (45.9)	2.0 (0.7;5.5)	1.7 (0.6;5.0)
60+	89 (20.8)	15 (40.5)	4.7 (1.6;13.5)*	2.3 (0.7;7.1)
**Overall knowledge**				
To a very high extent	6 (1.4)	2 (5.4)		
To a high extent	20 (4.7)	5 (13.5)		
Somewhat	57 (13.3)	14 (37.8)	6.9 (3.4;14.0)*	4.8 (2.2;10.4)*
To a lesser extent	143 (33.5)	11 (29.7)	1	1
Not at all	195 (45.7)	4 (10.8)		
Do not know/Not relevant	6 (1.4)	1 (2.7)		
**Overall attitude**				
Very positive	1 (0.2)	1 (2.7)		
Predominantly positive	24 (5.6)	7 (18.9)	3.8 (1.4;10.8)*	1.6 (0.5;5.1)
Neither positive nor negative	97 (22.7)	11 (29.7)	1	1
Predominantly negative	173 (40.5)	14 (37.8)	0.5 (0.2;1.1)	0.5 (0.2;1.1)
Very negative	119 (27.9)	3 (8.1)		
Don’t know/Not relevant	13 (3.0)	1 (2.7)		

Adjusted for: gender, age, practice type, overall knowledge of the GPs, and overall attitude of the GPs

^*^ Statistically significant at a *p*-value level ≤ 0.05

Twenty-one GPs, equal to more than half of the prescribers (56.8%) had quite some knowledge about MC and 15 of the prescribers (40.5%) had less or no knowledge of MC at all. A total of 17 prescribing GPs (45.9%) had a negative attitude towards MC, whereas 11 prescribers (29.7%) were neither positive nor negative, and 8 prescribers (21.6%) were positive towards it (see Table 2).

Most of the GPs stating to have quite some knowledge of MC were negative towards MC (50 GPs—60.3%), 16 GPs (19.3%) answered neutrally, and 15 GPs (18.1%) were positive towards MC (see Table 3).

**Table 3:** Associations between practice and GP characteristics and quite some knowledge of medicinal cannabis among GPs

	**All**	**Quite some knowledge of Medicinal Cannabis**	**Univariable regression**	**Multivariable regression**
	427 (100.0)	83 (100.0)		
**Practice type**				
Partnership	333 (78.0)	60 (72.3)	1	1
Singlehanded	94 (22.0)	23 (27.7)	1.3 (0.8;1.6)	0.8 (0.3;1.4)
**Gender**				
Female	236 (55.3)	25 (30.1)	1	1
Male	191 (44.7)	58 (69.9)	1.7 (1.5;1.8)*	1.7 (1.4;1.8)*
**Age**				
30–44	121 (28.3)	14 (16.9)	1	1
45–59	217 (50.8)	40 (48.2)	1.7 (0.9;3.3)	1.8 (0.9;3.5)
60+	89 (20.8)	29 (34.9)	3.7 (1.8;7.5)*	2.8 (1.3;6.0)*
**Overall attitude**				
Very positive	1 (0.2)	1 (1.2)		
Predominantly positive	24 (5.6)	14 (16.9)	7.7 (3.0;19.7)*	5.2 (1.9;14.0)*
Neither positive nor negative	97 (22.7)	16 (19.3)	1	1
Predominantly negative	173 (40.5)	36 (43.4)	1.1 (0.6;1.9)	1.0 (0.5;1.8)
Very negative	119 (27.9)	14 (16.9)		
Don’t know/Not relevant	13 (3.0)	2 (2.4)		

Adjusted for: gender, age, practice type and overall attitude of the GPs

^*^ Statistically significant at a *p*-value level ≤ 0.05

As for the GPs with a positive attitude towards MC, most of them stated to have quite some knowledge (15 GPs—60%) and 10 GPs (40%) to have less or no knowledge at all (see Table 4).

**Table 4:** Associations between practice and GP characteristics and positive attitudes among GPs towards medicinal cannabis

	**All**	**Positive Attitude**	**Univariable regression**	**Multivariable regression**
	427 (100.0)	25 (100.0)		
**Practice type**				
Partnership	333 (78.0)	16 (64.0)	1	1
Singlehanded	94 (22.0)	9 (36.0)	1.5 (0.9;1.8)	1.3 (0.1;1.7)
**Gender**				
Female	236 (55.3)	5 (20.0)	1	1
Male	191 (44.7)	20 (80.0)	1.8 (1.5;1.9)*	1.7 (1.1;1.9)*
**age**				
30–44	121 (28.3)	5 (20.0)	1	1
45–59	217 (50.8)	11 (44.0)	1.2 (0.4;3.7)	1.1 (0.4;3.5)
60+	89 (20.8)	9 (36.0)	2.6 (0.8;8.1)	1.4 (0.4;4.6)
**Overall knowledge**				
To a very high extent	6 (1.4)	1 (4.0)		
To a high extent	20 (4.7)	4 (16.0)		
Somewhat	57 (13.3)	10 (40.0)	7.4 (3.2;17.1)*	5.2 (2.2;12.5)*
To a lesser extent	143 (33.5)	6 (24.0)	1	1
Not at all	195 (45.7)	4 (16.0)		
Do not know/Not relevant	6 (1.4)			

Adjusted for: gender, age, practice type, and overall knowledge of the GPs

^*^ Statistically significant at a *p*-value level ≤ 0.05

### Results of the regression analyses

The odds of prescribing were higher when residing in singlehanded practices (OR=1.6, 95% CI 1.1;1.8) and almost five times higher when perceiving oneself to have quite some knowledge of MC (OR=4.8, 95% CI 2.2;10.4) (Table 2).

The odds of self-reported knowledge of MC were higher for male GPs (OR=1.7, 95% CI 1.4;1.8) and for the oldest age group (60 + years) (OR=2.8, 95% CI 1.3;6.0), compared to the youngest age group (30-44 years) (Table 3). In the group stating to be positive towards MC, the odds of reporting to have quite some knowledge was considerably higher than in the neutral and negative groups (OR=5.2, 95% CI 1.9;14.0).

Being male GP was significantly associated with a positive attitude towards use of MC and its prescription (OR=1.7, 95% CI 1.1;1.9), and the odds of having a positive attitude was more than five times higher when perceiving oneself to have quite some knowledge of MC (OR=5.2, 95% CI 2.2;12.5) (Table 4).

## Discussion

To our knowledge, this is the first study to explore GPs’ experience with prescription of MC and their knowledge, and attitudes towards prescribing it in a Danish setting. One and a half years after the launch of the national test scheme, 37 GPs (8.7% of the respondents) answered that they had prescribed MC to one or more patients and most of the GPs were males. Almost half of the prescribing GPs had a negative attitude towards MC and nearly one third were neither positive nor negative. Four out of five of the 427 responding GPs stated that they only had low or no knowledge of MC and more than two thirds were negative towards MC and its prescription.

Residing in a singlehanded practice and perceiving oneself to have quite some knowledge of MC were factors associated with prescription of MC. The odds of stating to have quite some knowledge of MC and its prescription were higher for males, in the 60+ age group, and in the group who stated to be positive towards MC. Being male and perceiving oneself to have quite some knowledge of MC was associated with a positive attitude towards MC, and prescription of it.

### Interpretation of the results

The finding from the regression analyses that GPs perceiving themselves to have quite some knowledge of MC was associated with a positive attitude towards MC and prescription of it was not surprising. Research has shown that physicians experienced in prescribing MC are more convinced of its benefits and less worried about adverse effects than physicians without these experiences [12, 25]. Conversely, the negative attitude among most GPs may be partially explained by the fact that they are responsible for prescribing MC according to Danish law, where some might fear the possibility for negative side effects [18].

Being a male GP was associated with a positive attitude towards MC as well. This finding is supported by previous studies in which being male was associated with early new drug prescription [26, 27]. In addition, we found that being a GP in a singlehanded practice was associated with prescription of MC. This finding is in contrast with findings from a previous study of diffusion of new drugs, which showed that partnership practices adopted new drugs faster than single-handed practices [28]. A possible explanation to our finding could be that the introduction of a new medicine in a partnership practice requires that everyone must agree on the use of it, before using that medicine in the practice. However, this study investigated medicine, which had been approved formally by authorities, and it may be different concerning non-approved medicine [28]. Previous literature has also found that physicians’ interest in particular therapeutic areas, participation in clinical trials, and volume of prescribing either in total or within the therapeutic class of the new drug, increases the likelihood of early adoption of new drugs [29].

The response rates were higher for GPs in the Central Denmark Region and the Region of Southern Denmark compared to The North Denmark Region, The Capital Region of Denmark and Region Zealand. This phenomenon is also observed in Danish GP surveys targeting other areas than medicinal cannabis [30, 31].

The low prescription rate of MC could be caused by a general prudence among GPs when new medicines or tools are introduced. This is for example seen in the introduction of video consultations in general practice [32]. The qualitative study on video consultations found three categories related to uncertainty about the new tool being 1) integrity, 2) setting, and 3) interaction. The uncertainties refer to 1) uncertainties related to how new technology may impede the provision of health care; 2) uncertainties related to the potentials of the technology; and 3) uncertainties related to how the technology affects interactions with patients. This can be transferred to uncertainties related to the prescription of MCs, namely the GPs' uncertainties about the potentials of cannabis as medicine [13, 32]. Besides from uncertainties related to prescription, the time aspect could also be an issue, given that it takes time to become familiar with a new type of medicine and the official guidelines that follow, in terms of indications recommended for prescription and use of available products on the market [19, 33].

The low prescription rate could, however, also be due to a perceived lack of training, as seen with the introduction of point-of-care ultrasound (POCUS) in family medicine [34]. POCUS’ ability to aid and guide in diagnosis and procedures has been demonstrated by numerous studies and it has been used for years by various medical specialties as a result [35, 36]. Many physicians working in family medicine did not feel they had the necessary training to begin with. They needed a training curriculum tailored to family medicine, which was later developed by colleagues within research. It lead to a significant improvement in confidence in their ability to perform and interpret a POCUS [34]. Implementation of newly established MC curriculums in general practice could also be a mean to improve confidence in usage among GPs, that in the end might have an effect on the present prescription rate [37, 38].

A recent systematic review that investigated physicians’ experiences, attitudes, and beliefs in MC found that a general lack of knowledge of clinical effects, both beneficial and adverse, affected their decision to prescribe [13]. Physicians from various specialties frequently experienced patient demands for MC, but their willingness to prescribe varied considerably. Hospital physicians and GPs experienced in prescribing were more convinced of effects and less worried about adverse effects. One way to increase the GPs’ knowledge and willingness to prescribe could be through already existing educational courses, as found in the UK and Denmark, that are based on the most recent evidence about treatment guidelines and the endocannabinoid system [39, 40].

### Limitations and strengths of the study

The response rate of our survey reached a total of 38.4% of the invited GPs which is comparable to response rates in similar studies [13]. Although, the proportion of GPs having prescribed MC and GPs with a positive attitude towards it is quite small, meaning that the results of the regression analyses, in which these variables are outcomes, only reveal tendencies along with actual associations.

The fact that the Danish Parliament has enacted a test scheme about MC prescription may have worried many physicians, as MC has not undergone the same rigorous clinical trial process as other new medications on the market and the physicians are responsible for prescribing it [41]. It is possible, that the most worried GPs (having the most negative attitudes) are also the ones who took the time to fill in the questionnaire to express their discontent with the test scheme. If so, it may have resulted in an overrepresentation of respondents with a negative attitude towards MC. However, it is also a possibility that the GPs with the most positive attitudes towards research in general are the ones responding to the questionnaire, regardless of their attitudes towards MC. This has been found in earlier patient studies [42, 43].

We did not perform a rigorous psychometric validation of the questionnaire. However, we carried out a thorough qualitative pilot test among five physicians in various relevant specialties focusing on content validity, relevance, acceptability, and feasibility. This led to some changes to the questionnaire. Hereafter, a small-scale quantitative pilot testing of the questionnaire was conducted among 12 physicians in relevant specialties with many GPs. Hence, we believe that the instrument and accordingly the results are reliable.

This cross-sectional study was conducted approximately one year after the four-year test scheme was enacted and provides knowledge of the factors influencing GPs’ decision to prescribe MC to patients, as well as their knowledge and attitudes towards it at this early point of time. We stress that prescribing patterns, knowledge, and attitudes may change throughout the remaining time of the test scheme, as they might have from the beginning to the end of our data collection. The long period of data collection was caused by a delay in data access from some regions.

## Conclusion

In this first study on prescription experience with MC, knowledge, and attitudes among Danish GPs, we find a very low proportion of prescribers, little knowledge, and an overall negative attitude towards MC. Among the prescribing GPs, almost half of them have little knowledge and a negative attitude towards MC. The GPs’ perception of having knowledge of MC is associated with their attitude towards it, and their decision to prescribe it to patients.

### Implications

In this study, we observe that the GPs’ prescribing patterns of MC are related to their attitudes and knowledge about it. Future studies should investigate the GPs’ prescribing patterns, experience, and attitudes in other similar sized countries with differing MC policies to check if similar or differing patterns emerge, preferably using the same or a similar questionnaire for further validation. Future studies should also investigate the influence of patient-reported effects and side effects on the GPs’ prescription patterns, as this might also play a role in their decision to prescribe. Furthermore, it could be interesting to look at how often GPs are renewing patients’ MC prescriptions, to clarify whether patients have positive experiences with MC. Finally, it could also be interesting to look at GPs’ substitution of patients’ opioid prescriptions with MC prescriptions, to clarify whether GPs and patients prefer MC over opioids.

## Supplementary Information

**Additional file 1: Appendix. **Translated English version of the Danish national GP questionnaire: “Physicians’ experience, knowledge, and attitudes to medicinal cannabis”*.*

## Data Availability

The datasets generated and analyzed in the current study are available from the corresponding author on reasonable request, but restrictions apply to the availability of these data due to the data protection regulations from the Danish Data Protection Agency, and so are not publicly available. Access to data is strictly limited to the researchers who have obtained permission for data processing. This permission was given to the Research Unit of General Practice, Department of Public Health, University of Southern Denmark.

## Abbreviations

GP: General practitioner
MC: Medicinal cannabis
CBM: Cannabis-based medicine
OR: Odds ratio
