# Supplementary material for: GPs’ prescription patterns, experience, and attitudes towards medicinal cannabis—a nationwide survey at the early stage of the Danish test scheme
Source: BMC Prim Care. 2023 Jan 17;24:17. doi: 10.1186/s12875-023-01971-4 (PMC9843989; doi:10.1186/s12875-023-01971-4)
Supplement: Supplementary file 1 — Additional file 1: Appendix. Translated English version of the Danish national GP questionnaire: “Physicians’ experience, knowledge, and attitudes to medicinal cannabis”. [file 12875_2023_1971_MOESM1_ESM.docx]

**Appendix**

*Translated English version of the Danish national GP questionnaire: “Physicians’ experience, knowledge, and attitudes to medicinal cannabis”*

| **Physicians’ experience, knowledge, and attitudes to medicinal cannabis** |
| --- |

Thank you for taking the time to answer the questionnaire. You must answer all questions on one page to move on to the next page. You can always go back to previous answers and change them if you need to.

To move forward in the questionnaire, click the "Next" button. If you wish to change your answers, please use the "Previous" button.

| **In the following, the questions are about your experience with prescribing medicinal cannabis and cannabis-based medicines**  **Medicinal cannabis** is plant parts or extracts of plant parts (e.g., oil) from the cannabis plant and is not approved as a medicine in Denmark. This medicine became available with the passage of the four-year trial scheme bill, which came into effect on 1 January 2018, e.g., Bedrocan and Bediol.  **Cannabis-based medicines** are approved medicines based on extracts of the cannabis plant or synthetically produced cannabinoids (CBD and THC), e.g., Sativex and Nabilone. |
| --- |

**Do you have experience in prescribing medicinal cannabis and/or cannabis-based medicines?**

**You may tick off more than one**

(1) ❑ Yes, I have prescribed medicinal cannabis to one or more patients

(2) ❑ Yes, I have prescribed cannabis-based medicines to one or more patients

(3) ❑ No, I have never prescribed medicinal cannabis or cannabis-based medicines to patients

How many patients have you prescribed medicinal cannabis to, since it became possible after 1 January 2018?

(1) ❑ 1-3

(2) ❑ 4-6

(3) ❑ 7-10

(4) ❑ 11-20

(5) ❑ 21-30

(6) ❑ 31 or more

|  |
| --- |

**For which indications have you prescribed medicinal cannabis? You may tick off more than one**

(1) ❑ Neurogenic pain

(2) ❑ Neuropathic pain

(3) ❑ Pain in several places in the musculoskeletal system, including fibromyalgia, Bodily Distress Syndrome, musculoskeletal type, and others.

(4) ❑ Pain in chronic rheumatoid arthritis

(5) ❑ Pain in psoriatic arthritis

(6) ❑ Pain in spondyloarthritis (Bechterew's disease)

(7) ❑ Pain from osteoarthritis (osteoarthritis)

(8) ❑ Pain from brittle bones (osteoporosis)

(9) ❑ Spasticity due to multiple sclerosis

(10) ❑ Central nerve pain due to spinal cord injury (paraplegia)

(11) ❑ Spasticity due to spinal cord injury (paraplegia)

(12) ❑ Cancer pain

(13) ❑ Nausea and vomiting after chemotherapy

(14) ❑ Other disorders ________________________________________

**Which of the following cannabis preparations have you prescribed under the trial scheme?**

**You may tick off more than one**

(4) ❑ Bedrocan "Canngros"

(5) ❑ Bediol "Canngros"

(7) ❑ Bedica "Canngros"

(8) ❑ 1:1 Drops "Stenocare"

(9) ❑ CBD Drops "Stenocare"

(10) ❑ THC Drops "Stenocare"

**Have you experienced side effects in your patients that you attribute to treatment with medicinal cannabis?**

(1) ❑ Yes

(2) ❑ No

**Have you experienced the following side effects in your patients that you attribute to treatment with**

**medicinal cannabis? You may tick off more than one**

(1) ❑ Fatigue

(2) ❑ Changed mood

(3) ❑ Dizziness

(4) ❑ Anxiety

(5) ❑ Influence of concentration

(6) ❑ Influence of memory

(7) ❑ Drop in blood pressure leading to dizziness and possibly fainting

(8) ❑ Liver influence

(9) ❑ Depression

(10) ❑ Hallucinations

(11) ❑ Paranoia

(12) ❑ Delusions

(13) ❑ Suicidal thoughts

(14) ❑ Dependence

(15) ❑ Other side effects? _____

**To how many patients have you prescribed cannabis-based medicines?**

(1) ❑ 1-3

(2) ❑ 4-6

(3) ❑ 7-10

(4) ❑ 11-20

(5) ❑ 21-30

(6) ❑ 31 or more

**Which indications made you prescribe cannabis-based medicines? You may tick off more than one**

(1) ❑ Neurogenic pain

(2) ❑ Neuropathic pain

(3) ❑ Pain in several places in the musculoskeletal system, including fibromyalgia, Bodily Distress Syndrome, musculoskeletal type, and others.

(4) ❑ Pain in chronic rheumatoid arthritis

(5) ❑ Pain in psoriatic arthritis

(6) ❑ Pain in spondyloarthritis (Bechterew's disease)

(7) ❑ Pain from osteoarthritis (osteoarthritis)

(8) ❑ Pain from brittle bones (osteoporosis)

(9) ❑ Spasticity due to multiple sclerosis

(10) ❑ Central nerve pain due to spinal cord injury (paraplegia)

(11) ❑ Spasticity due to spinal cord injury (paraplegia)

(12) ❑ Cancer pain

(13) ❑ Nausea and vomiting after chemotherapy

(14) ❑ Other disorders ________________________________________

**Which of the following cannabis-based medicines have you prescribed? You may tick off more than one**

(1) ❑ Sativex

(2) ❑ Marinol

(3) ❑ Nabilone

(6) ❑ Magisterially produced cannabis

**Have you experienced side effects in your patients that you attribute to treatment with cannabis-based**

**medicines?**

(1) ❑ Yes

(2) ❑ No

**Have you experienced the following side effects in your patients that you attribute to treatment with cannabis-based medicines? You may tick off more than one**

(1) ❑ Fatigue

(2) ❑ Changed mood

(3) ❑ Dizziness

(4) ❑ Anxiety

(5) ❑ Influence on concentration

(6) ❑ Influence on memory

(7) ❑ Drop in blood pressure leading to dizziness and possible fainting

(8) ❑ Influence on liver

(9) ❑ Depression

(10) ❑ Hallucinations

(11) ❑ Paranoia

(12) ❑ Delusions

(13) ❑ Suicidal thoughts

(14) ❑ Dependence

(15) ❑ Other side effects? _____

Which of the following factors influence your decision to prescribe medicinal cannabis to your patients?

|  | To a very high extent | To a high extent | Somewhat | To a lesser extent | Not at all | Do not know/not relevant |
| --- | --- | --- | --- | --- | --- | --- |
| The severity of the patient's illness | (1) ❑ | (2) ❑ | (3) ❑ | (4) ❑ | (5) ❑ | (9) ❑ |
| That I have experienced a good effect of medicinal cannabis in patients | (1) ❑ | (2) ❑ | (3) ❑ | (4) ❑ | (5) ❑ | (9) ❑ |
| That the patient has previously tried other medicinal treatments and different dosages thereof without sufficient effect | (1) ❑ | (2) ❑ | (3) ❑ | (4) ❑ | (5) ❑ | (9) ❑ |
| That I have a mutually trusting relationship with the patient | (1) ❑ | (2) ❑ | (3) ❑ | (4) ❑ | (5) ❑ | (9) ❑ |
| That the patient wants it | (1) ❑ | (2) ❑ | (3) ❑ | (4) ❑ | (5) ❑ | (9) ❑ |
| That the patient turns to the doctor to get an assessment of the relevance of medicinal cannabis in relation to the current disorder | (1) ❑ | (2) ❑ | (3) ❑ | (4) ❑ | (5) ❑ | (9) ❑ |
| Other conditions. Specify which: | (1) ❑ | (2) ❑ | (3) ❑ | (4) ❑ | (5) ❑ | (9) ❑ |

**Have you experienced a change in the number of inquiries regarding medicinal cannabis in connection with the introduction of the new trial scheme?**

(1) ❑ Yes, an increase

(2) ❑ Yes, a fall

(3) ❑ No, no change

(4) ❑ There have been no inquiries at all

**What purpose(s) did the patients have with these inquiries? You may tick off more than one**

(1) ❑ They would investigate the possibility of having medicinal cannabis prescribed for their illness/disorder

(2) ❑ They were interested because of the media exposure of the subject

(3) ❑ They sought to legalize an already existing use of medicinal cannabis

(4) ❑ Other(s) _____

**On whose initiative do you typically talk to your patients about the possibility of prescribing**

**medicinal cannabis? You may tick off more than one**

**I typically talk to my patients about prescribing medicinal cannabis on:**

(1) ❑ The patient's initiative

(2) ❑ My own initiative

(3) ❑ Another treatment center's initiative

(4) ❑ Initiative of the patient's relatives

(5) ❑ Others' initiative. State if: _____

**What do you pay most attention to when talking to your patients about medicinal cannabis?**

**You may tick off maximum 3**

(1) ❑ The evidence in the field

(2) ❑ To eliminate misconceptions regarding the effect of medicinal cannabis

(3) ❑ Indication

(4) ❑ Possible effects of the treatment

(5) ❑ Other treatment options

(6) ❑ Side effects/risks of the treatment

(7) ❑ The legal responsibility for the treatment

(8) ❑ Price and subsidy

(9) ❑ Other things _____

Do you or your relatives have experience of serious illness?

(1) ❑ Yes

(2) ❑ No

(3) ❑ Do not want to answer

**Have you or any of your relatives used medicinal cannabis or cannabis-based medicines in connection**

**with the disease in question? You may tick off more than one**

(1) ❑ Yes, medicinal cannabis

(4) ❑ Yes, cannabis-based medicines

(2) ❑ No, none of the above

(3) ❑ Do not want to answer

**How was your experience?**

(1) ❑ Very positive

(2) ❑ Predominantly positive

(3) ❑ Neither positive nor negative

(4) ❑ Predominantly negative

(5) ❑ Very negative

(6) ❑ Do not know/not relevant

**If you have comments on this part of the questionnaire, please write them here:**

_____________________________________________
_____________________________________________
_____________________________________________
_____________________________________________
_____________________________________________
_____________________________________________

| **In the following, the questions are about your knowledge of medicinal cannabis** |
| --- |

**To what extent do you feel that you have sufficient knowledge of medicinal cannabis to prescribe it?**

(1) ❑ To a very high extent

(2) ❑ To a high extent

(3) ❑ Somewhat

(4) ❑ To a lesser extent

(5) ❑ Not at all

(9) ❑ Do not know/not relevant

To what extent do you feel that you have sufficient knowledge of the following matters in relation to prescribing medicinal cannabis?

|  | To a very high extent | To a high extent | Somewhat | To a lesser extent | Not at all | Do not know/not relevant |
| --- | --- | --- | --- | --- | --- | --- |
| Indications | (1) ❑ | (2) ❑ | (3) ❑ | (4) ❑ | (5) ❑ | (9) ❑ |
| Dosing | (1) ❑ | (2) ❑ | (3) ❑ | (4) ❑ | (5) ❑ | (9) ❑ |
| Dosage form | (1) ❑ | (2) ❑ | (3) ❑ | (4) ❑ | (5) ❑ | (9) ❑ |
| Side effects | (1) ❑ | (2) ❑ | (3) ❑ | (4) ❑ | (5) ❑ | (9) ❑ |
| Addiction | (1) ❑ | (2) ❑ | (3) ❑ | (4) ❑ | (5) ❑ | (9) ❑ |
| Rules for driving after ingestion | (1) ❑ | (2) ❑ | (3) ❑ | (4) ❑ | (5) ❑ | (9) ❑ |
| Risks of serious side effects, e.g., psychosis | (1) ❑ | (2) ❑ | (3) ❑ | (4) ❑ | (5) ❑ | (9) ❑ |
| The legal responsibility for the processing | (1) ❑ | (2) ❑ | (3) ❑ | (4) ❑ | (5) ❑ | (9) ❑ |
| Evidence | (1) ❑ | (2) ❑ | (3) ❑ | (4) ❑ | (5) ❑ | (9) ❑ |
| Effect | (1) ❑ | (2) ❑ | (3) ❑ | (4) ❑ | (5) ❑ | (9) ❑ |
| Price | (1) ❑ | (2) ❑ | (3) ❑ | (4) ❑ | (5) ❑ | (9) ❑ |
| Subsidy for the patient/from the health insurance | (1) ❑ | (2) ❑ | (3) ❑ | (4) ❑ | (5) ❑ | (9) ❑ |
| Administration | (1) ❑ | (2) ❑ | (3) ❑ | (4) ❑ | (5) ❑ | (9) ❑ |

**If you have comments on this part of the questionnaire, please write them here:**

_____________________________________________
_____________________________________________
_____________________________________________
_____________________________________________
_____________________________________________
_____________________________________________

| **In the following, the questions are about your attitude towards medicinal cannabis** |
| --- |

**What is your position on prescribing medicinal cannabis?**

(1) ❑ Very positive

(2) ❑ Predominantly positive

(3) ❑ Neither positive nor negative

(4) ❑ Predominantly negative

(5) ❑ Very negative

(6) ❑ Do not know/not relevant

**Have you adopted a policy on prescribing medicinal cannabis in your practice/in your department?**

(1) ❑ Yes

(2) ❑ No

**What is the adopted policy in your practice/at your department?**

________________________________________
________________________________________
________________________________________
________________________________________
________________________________________

To what extent do you agree to the following statements?
Medicinal cannabis is relevant in the treatment of:

|  | Strongly agree | Mostly agree | Neither agree nor disagree | Mostly disagree | Strongly disagree | Do not know/not relevant |
| --- | --- | --- | --- | --- | --- | --- |
| Neurogenic pain | (1) ❑ | (2) ❑ | (3) ❑ | (4) ❑ | (5) ❑ | (6) ❑ |
| Neuropathic pain | (1) ❑ | (2) ❑ | (3) ❑ | (4) ❑ | (5) ❑ | (6) ❑ |
| Pain in several places in the musculoskeletal system, including fibromyalgia, Bodily Distress Syndrome, musculoskeletal type and others. | (1) ❑ | (2) ❑ | (3) ❑ | (4) ❑ | (5) ❑ | (6) ❑ |
| Pain in chronic rheumatoid arthritis | (1) ❑ | (2) ❑ | (3) ❑ | (4) ❑ | (5) ❑ | (6) ❑ |
| Pain in psoriatic arthritis | (1) ❑ | (2) ❑ | (3) ❑ | (4) ❑ | (5) ❑ | (6) ❑ |
| Pain in spondyloarthritis (Bechterew's disease) | (1) ❑ | (2) ❑ | (3) ❑ | (4) ❑ | (5) ❑ | (6) ❑ |
| Pain from osteoarthritis (osteoarthritis) | (1) ❑ | (2) ❑ | (3) ❑ | (4) ❑ | (5) ❑ | (6) ❑ |
| Spasticity due to multiple sclerosis | (1) ❑ | (2) ❑ | (3) ❑ | (4) ❑ | (5) ❑ | (6) ❑ |
| Central nerve pain due to spinal cord injury (paraplegia) | (1) ❑ | (2) ❑ | (3) ❑ | (4) ❑ | (5) ❑ | (6) ❑ |
| Painful spasticity due to spinal cord injury (paraplegia) | (1) ❑ | (2) ❑ | (3) ❑ | (4) ❑ | (5) ❑ | (6) ❑ |
| Cancer pain | (1) ❑ | (2) ❑ | (3) ❑ | (4) ❑ | (5) ❑ | (6) ❑ |
| Nausea and vomiting after chemotherapy | (1) ❑ | (2) ❑ | (3) ❑ | (4) ❑ | (5) ❑ | (6) ❑ |
| Other disorders. Specify which: | (1) ❑ | (2) ❑ | (3) ❑ | (4) ❑ | (5) ❑ | (6) ❑ |

_____________________________________________

How much do you agree with the statements below?

|  | Strongly agree | Mostly agree | Neither agree nor disagree | Mostly disagree | Strongly disagree | Do not know/not relevant |
| --- | --- | --- | --- | --- | --- | --- |
| Medicinal cannabis is too expensive for patients | (1) ❑ | (2) ❑ | (3) ❑ | (4) ❑ | (5) ❑ | (6) ❑ |
| There is sufficient evidence for the effectiveness of treatment with medicinal cannabis | (1) ❑ | (2) ❑ | (3) ❑ | (4) ❑ | (5) ❑ | (6) ❑ |
| The possibility of legally using medicinal cannabis should benefit patients regardless of the evidence base | (1) ❑ | (2) ❑ | (3) ❑ | (4) ❑ | (5) ❑ | (6) ❑ |
| Medicinal cannabis should mainly be used in palliative care | (1) ❑ | (2) ❑ | (3) ❑ | (4) ❑ | (5) ❑ | (6) ❑ |
| Medicinal cannabis should only be used when other existing treatment methods have not had sufficient effect | (1) ❑ | (2) ❑ | (3) ❑ | (4) ❑ | (5) ❑ | (6) ❑ |
| Medicinal cannabis should only be used when other existing treatment methods have not had sufficient effect | (1) ❑ | (2) ❑ | (3) ❑ | (4) ❑ | (5) ❑ | (6) ❑ |
| Medicinal cannabis should go through the same approval procedure as all medicines | (1) ❑ | (2) ❑ | (3) ❑ | (4) ❑ | (5) ❑ | (6) ❑ |
| It should be a medicinal task to legalize patients' existing use of medicinal cannabis | (1) ❑ | (2) ❑ | (3) ❑ | (4) ❑ | (5) ❑ | (6) ❑ |
| The expertise of doctors should have been involved to a greater extent in connection with the introduction of the trial scheme with medicinal cannabis | (1) ❑ | (2) ❑ | (3) ❑ | (4) ❑ | (5) ❑ | (6) ❑ |
| The trial scheme increases the risk of harming the patient | (1) ❑ | (2) ❑ | (3) ❑ | (4) ❑ | (5) ❑ | (6) ❑ |

**How concerned are you about the resale of legally prescribed medicinal cannabis?**

(1) ❑ To a very high extent

(2) ❑ To a high extent

(3) ❑ Somewhat

(4) ❑ To a lesser extent

(5) ❑ Not at all

(9) ❑ Do not know/not relevant

**Do you know whether your patients buy/have bought cannabis for medicinal use on the black market?**

(1) ❑ Yes

(2) ❑ No

**How much do you agree that cannabis should be legalized in general?**

(1) ❑ Strongly agree

(2) ❑ Mostly agree

(3) ❑ Neither agree nor disagree

(4) ❑ Mostly disagree

(5) ❑ Strongly disagree

(6) ❑ Do not know

**If you have comments on this part of the questionnaire, please write them here:**

_____________________________________________
_____________________________________________
_____________________________________________
_____________________________________________
_____________________________________________
_____________________________________________

| **In the following, the questions are about yourself and your work** |
| --- |

**What specialty are you trained in?**

(1) ❑ General medicine

(2) ❑ Anaesthesia

(3) ❑ Neurology

(4) ❑ Oncology

**How long have you worked in your specialty?**

(1) ❑ Less than a year

(2) ❑ A year or more

**State how many years you have worked in your specialty**

__

**Do you work as a general practitioner?**

(1) ❑ Yes

(2) ❑ No

Is your practice?

(1) ❑ Singlehanded

(2) ❑ Partnership

**Is your practice part of a collaborative practice?**

(1) ❑ Yes

(2) ❑ No

**Are you in a shared practice?**

(1) ❑ Yes

(2) ❑ No

**What year did you start in general practice?**

____

**What specialization do you work in?**

________________________________________

**Which department do you work in?**

________________________________________

What is your age?

__

Are you?

(1) ❑ Male

(2) ❑ Female

**If you have comments on the questionnaire in general, or there are other things than those already mentioned that you consider important in connection with the experimental scheme with medicinal cannabis, you are welcome to write them here:**

_____________________________________________
_____________________________________________
_____________________________________________
_____________________________________________
_____________________________________________
_____________________________________________

**To facilitate contact with you, we would like to be allowed to contact you by email in connection with this or future projects. Your commitment can be withdrawn at any time. The information will not be passed on to third parties.**

________________________________________
________________________________________
________________________________________
________________________________________
________________________________________

| You have now finished your answer.  If you want to print it out, press the print icon below.  To end the survey, press the "Finish" button.  Thank you for your participation! |
| --- |
